# Clinical Evidence in the Treatment of Obstructive Sleep Apnoea with Oral Appliances: A Systematic Review

**DOI:** 10.1155/2021/6676158

**Published:** 2021-05-08

**Authors:** Andrea Rossi, Antonino Lo Giudice, Camilla Di Pardo, Alberto Teodoro Valentini, Francesca Marradi, Nicola Vanacore, Cristina Grippaudo

**Affiliations:** ^1^Department of Head and Neck Surgery, Fondazione Policlinico Gemelli IRCCS, Dental Institute, Catholic University of Sacred Heart, Rome, Italy; ^2^Department of General Surgery and Medical-Surgical Specialties, Section of Orthodontics, School of Dentistry, University of Catania, Catania, Italy; ^3^Department of Medicine and Aging Sciences, “G. d'Annunzio” University of Chieti-Pescara, Chieti, Italy; ^4^National Center for Disease Prevention and Health Promotion, Italian National Institute of Health, Rome, Italy

## Abstract

**Background:**

Recent clinical guidelines have extended indications for oral appliances to subjects affected by moderate-to-severe obstructive sleep apnoea (OSA). The aim of this systematic review covering this important issue for public health is twofold: updating and summarizing the best available scientific evidence by selecting RCTs of quality only, and identifying the therapeutic pathways that can be transferred to the current clinical practice.

**Methods:**

All the abstracts which were published before February 18, 2019, have been identified in three electronic databases (PubMed, Web of Science, and Cochrane). The Cochrane Collaboration's tool for assessing risk of bias was used as an assessment tool in order to evaluate the quality of the selected studies.

**Results:**

The search strategy yielded 2,260 studies. After removing duplicates and studies that did not comply with the inclusion criteria, 32 full-text articles were assessed for eligibility, and 17 RCTs were finally included in the qualitative synthesis. The 17 selected studies were very heterogeneous in the type of included RCTs in terms of patient inclusion criteria, sample size, distribution of the two genders in the various groups, duration of treatment, and definition of primary and secondary outcomes, without any restriction on the definition of the control group. A common finding was the positive responsiveness of oral appliance treatment in subjects affected by mild-to-moderate OSA with some evidence for cases of severe OSA.

**Conclusion:**

Higher-quality studies are needed in order to provide additional useful guidelines for dental clinicians for OSA management.

## 1. Background

In a recent systematic review, Senaratna et al., highlighted the fact that the overall prevalence of any OSA ranged from 9% to 38% in the general adult population, from 13% to 33% in men and from 6% to 19% in women. OSA prevalence was much higher in older ages, in males, and in those with higher BMI. [1]. The prevalence of moderate-to-severe sleep-disordered breathing (AHI ≥15 events per h) in a general population of predominantly white European origin with a median age of 57 years was estimated to be 23.4% in women and 49.7% in men. [2]. OSA increases the risk of hypertension, glucose intolerance, and cardiovascular and cerebrovascular disorders. Untreated OSA is also associated with daytime sleepiness, cognitive dysfunction, and increased risk of road accidents [3]. OSA syndrome can be treated with different types of surgical or nonsurgical therapeutic approaches [4]. Oral appliances treatment for OSA is widely used in mild-to-moderate OSA forms [5]. Dentists are increasingly being consulted in the multidisciplinary treatment of snoring and sleep apnoea/hypopnoea syndrome. The most recent clinical guidelines have also extended indications for oral appliances to moderate and severe OSA cases when a patient, after having been duly informed about the risks, refuses the CPAP (continuous positive airway pressure) treatment [6]. Currently, there are around 100 devices available for OSA treatment [7]. These devices can be mainly distinguished in three categories: mandibular advancement devices, tongue-retaining devices, and soft palate-lifting devices [8, 9]. Several systematic reviews on the efficacy of oral appliances (OA) for OSA treatment were published over the past years [9–16]. These systematic reviews were published from 2006 to 2019 and were based on articles published before 2018 [16]. Six revisions only include randomised clinical trials (RCT) [9–12, 14, 15], one includes randomised and nonrandomised clinical trials [13], and the other one includes RCT and observational studies [16]. All of these systematic reviews include a single RCT without any limitation in the number of sample sizes with a range from a few dozens of patients to less than one hundred patients. These systematic reviews assessed OA effectiveness compared to CPAP [15], placebo device or nontreated subjects [13], other types of OA [9, 10], and other interventions (medical and surgical treatments) [11, 12, 14, 16]. These studies have significant clinical heterogeneity in terms of sample size, follow-up, and identified endpoints. Finally, the quality checklists for RCTs (Jadad score and Cochrane tool for risk of bias) were only used in three systematic revisions [11, 13, 15].

The aim of this systematic review covering an important issue for public health is twofold: updating and summarizing the best available scientific evidence by only selecting RCTs of quality, and identifying the therapeutic pathways that can be transferred to the current clinical practice.

## 2. Materials and Methods

A systematic review based on the PRISMA (Preferred Reporting Items for Systematic Reviews and Meta-Analyses) checklist was carried out [17]. All abstracts which were published before February 18, 2019, have been identified in three electronic databases (PubMed, Web of Science, and Cochrane Library). The following search strategies were adopted: “obstructive sleep apnoea” OR “obstructive sleep apnoea” OR “obstructive sleep apnoeas” OR “obstructive sleep apnoeas” AND “mandibular advancement device” OR “mandibular advancement devices” OR “dental device” OR “dental devices” OR “dental appliance” OR “dental appliances” OR “oral device” OR “oral devices” OR “oral appliance” OR “oral appliances”. Selected studies had to be of RCT type and investigate the efficacy of oral appliances and/or analyse potential positive or negative predictive factors in OSA treatment. Articles were selected by searching the results obtained from the three databases.

The following inclusion criteria were adopted:

- RCTs evaluating OA vs other treatments (surgical or nonsurgical approaches) which included at least a total of 50 patients
- Subjects' age ≥18 years
- Studies in English
- Studies comparing several oral appliances
- Studies comparing the oral appliance treatment to placebo devices; nontreated control groups; patients treated with other therapeutic approaches for OSA (CPAP, surgery, positional treatment); and a combination of the above comparisons

The exclusion criteria were as follows:

- Studies with biological markers as the primary endpoint
- Observational studies (case-control and cohort studies)
- Case reports
- Case series
- Systematic reviews and meta-analyses
- Animal studies
- Secondary RCTs (studies with secondary analysis compared to the primary endpoint of the trial)

Two reviewers selected the studies in duplicate and independently, in accordance with the inclusion criteria, with the review process being double blind. When the two reviewers could not reach an agreement on the selection of an article, a third expert evaluator was called to end the dispute.

The primary outcome was represented by the improvement of respiratory indices (AHI: apnoea-hypopnoea index; RDI: respiratory disturbance index) because these indices represent the most used assessment tools in evaluating the success of OSA treatment in the scientific literature. The secondary outcomes of this review were the following: positive predictive factors in OA for OSA treatment; daytime sleepiness (ESS), quality of life, compliance, and snoring. A specific extraction table was used to summarize the content of each study. The extraction table was divided into the following sections: bibliographical references; RCT design and type of comparison, sample size, type of oral appliance, follow-up, and main conclusion on the primary outcome.

Cochrane Collaboration's tool for assessing risk of bias was used to assess the quality of the studies [18]. This checklist assesses seven domains of a study (random sequence, allocation concealment, blinding of participants and personnel, blinded outcome assessment, incomplete outcome data, selective reporting, and other bias) and the overall risk of bias.

## 3. Results

**Figure 1:**
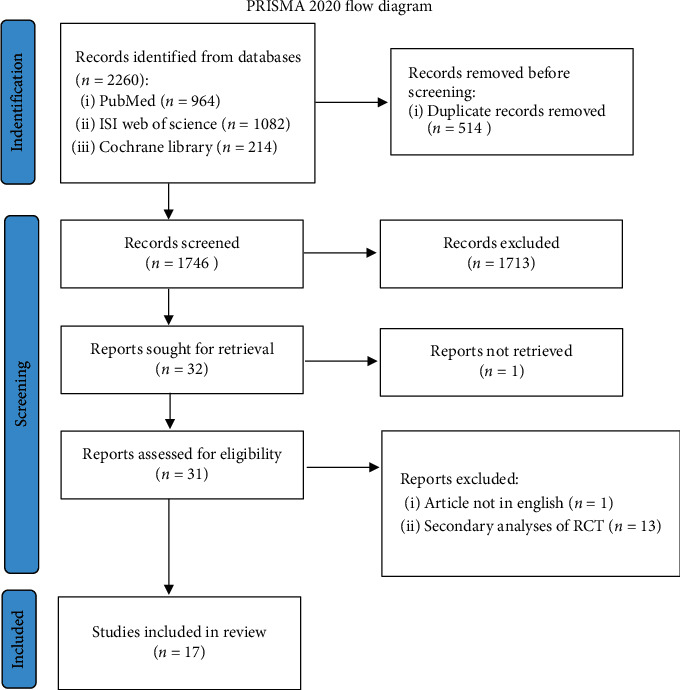
PRISMA 2020 flow diagram.

The process of literature search and selection is displayed in Figure 1. The search strategy yielded 2,260 studies. After removing duplicates and studies that did not comply with the inclusion criteria, 32 full-text articles were assessed for eligibility, and finally 17 RCTs were included in the qualitative synthesis.

The 17 selected studies were very heterogeneous. A first important difference that had not been taken into account in the previous systematic reviews was the use of different types of oral appliances in these studies (Table 1). Banhiran et al. [19] used a noncustomised oral appliance. In 6 studies a customised, but not titratable, device was used [20–25]. In their study, Quinnel et al. [22] compared a nontitratable, customised device with two other appliances: a customised appliance made by a dental technician on models realised from dental records worn by patients with a specific kit and another preformed device (“boil and bite”). Other studies used a customised and titratable appliance [26–35].

Another important difference in these studies can be noted by observing the different duration of the follow-up periods (from 1 to 42.7 months) and the different severity of the respiratory index of the included patients (Table 1). The sample size of 17 RCTs is included between 50 and 150 patients (Table 1).

Among the selected 6 studies comparing the oral appliance treatment with CPAP [20, 26, 27, 29, 33, 35], only 2 studies included a placebo group [26, 27]. In one of these studies [20], a third group was only treated with conservative measures (tips for sleep hygiene and diet in overweight subjects). In 5 studies, the oral appliance treatment was only compared with placebo [21, 25, 30, 32, 34]. In the study by Banhiran et al. [19], there is not a placebo group, and a thermoplastic noncustomised titratable device is used (as opposed to what is suggested by the current guidelines). A certain degree of heterogeneity was also noted among the placebo treatments. Four studies used various types of upper-jaw splint [26, 30, 32, 34], one study used a placebo tablet [27], and another one used the same appliance of the active group without mandibular advancement [25]. Two studies compared several types of mandibular advancement devices [22, 31], and only one of these studies had a no-treatment control group [31]. Only one study compared different degrees of mandibular advancement [23]. Finally, a study compared the surgical approach (UPPP) with the oral appliance treatment [24], and one of the 17 studies compared the positional treatment with the oral appliance treatment [28]. None of these studies had a placebo group. As shown in Table 1, among the 17 selected RCTs, 9 RCTs had a parallel design and 8 RCTs had a crossover design. Only three studies were noninferiority RCTs (Table 1).

Each one of the studies was analysed, and the primary and secondary outcomes were identified. For each article, the presence of secondary RCTs among the previously excluded full-text articles was also analysed [36–48]. The quality assessment of 17 RCTs showed that only two studies have an overall risk of bias defined as “low risk” [25, 26] (Table 2).

## 4. Discussion

This systematic review took into account the evidence published until February 2019 and showed high heterogeneity in the type of included RCTs in terms of patient inclusion criteria, sample size, distribution of the two genders in the various groups, treatment duration, and definition of primary and secondary outcomes without any restriction on the definition of the control group. In this systematic review, the therapeutic success of OA for OSA treatment was evaluated in accordance with the American Academy of Sleep Medicine (AASM) efficacy criteria (partial success, AHI reduced by 50% if compared to AHI at baseline with a value ≥5; complete success: AHI value less than 5), and only RCTs with more than 50 patients were included. Moreover, the Cochrane Collaboration's tool for assessing risk of bias was used in order to assess the quality of the RCT. Further consideration should be given to heterogeneity: the type of oral appliance that must be chosen. No systematic review carefully considered this element in the assessment of the effectiveness and level of compliance of the patients. In our study, 10 of the 17 selected RCTs (58.8%) have taken into account customised and titratable devices, allowing high customisation of the treatment.

Currently, there is a guidelines' reference document published in 2015 by the American Academy of Sleep Medicine (AASM) and the American Academy of Dental Sleep Medicine (AADSM) in the dental clinical practice for the OSA treatment. This document clarifies the importance of customised and titratable oral appliances in the OSA treatment [6]. As shown by the use of the Cochrane Collaboration's tool for assessing risk of bias, the overall quality of the selected studies is low, and only two studies have an overall risk of bias defined as “low risk” [25, 26]. It is noticeable that 29.4% of the studies [22, 25, 27, 31, 34] did not carry out a sample analysis of the trial if compared to a priori efficacy priority of the treatment stating that there was a possibility of being wrong about the false negative (power of the study) and the false positive (*p* value). The lack of this information has significant implications not only for the quality of the results, but also from an ethical point of view. Moreover, 6 out of 17 articles (35.3%) [23, 24, 27, 30–32] lack the information related to randomisation and blindness. If the study is blinded, the subjects who are blinded after the allocation to the intervention group (for example, professional participants delivering support, results' evaluators) and the modality make the evidence extremely likely to be criticised. For these reasons, there is a lack of clinical and methodological assumptions to carry out our meta-analysis. Better-quality studies will hopefully be conducted in the future with an improved quality of heterogeneous clinical criteria in order to appropriately use a meta-analytical study design.

The primary outcome for many studies was the polysomnographic parameters evaluation and the improvement of respiratory indices. Among these studies, there was the one by Aarab et al. [26] showing a lack of statistically significant differences between the CPAP treatment group and the mandibular advancement device (MAD) group in AHI improvement. In this study, there is a problem related to a significant statistical difference of the BMI at baseline between the two groups (CPAP and MAD). The medium baseline body mass index (BMI) in the MAD group was lower than the one in the CPAP group. Notwithstanding, the difference, the increase in weight, is a factor which is strictly connected to the seriousness of apnoea, and in light of the current evidence in OA treatment, the results of the study can be considered reliable. The only sleep parameter which has significantly improved in the statistics both with CPAP and MAD compared to placebo was the RAI (respiratory arousal index) parameter. Among the best qualitative studies comparing OA and CPAP, Aarab et al. [26] had the longest follow-up period. In addition to Aarab et al. [26], there was another study comparing CPAP and OA published by Lam et al. [20] in which there was not a placebo group, but there was a third conservative treatment group (diet and weight control). The available data from this study outlined better results with CPAP and slightly lower results with MAD, showing that weight control could lead to better sleep parameters, but these parameters were not heterogeneous and statistically significant compared to baseline. CPAP proved to be better in terms of efficacy compared to MAD that showed an increased tolerability level and a better compliance from patients.

From the assessment of the 17 selected studies and their secondary publications, the following considerations on the oral appliance treatment for OSA and snoring resulted:

- OAs were efficient in reducing the AHI with an average of more than 50% compared to baseline also in subjects with severe OSA reaching a partial success [30]. In the same way, OA treatment was able to reduce the ODI median (3%) by half in these subjects [30]. In this study [30], it is also interesting to notice that the AI value (apnoea index) reached a median value of 1 [0–5] from an average of 8.5 showing a great level of success in solving apnoea and a prevalence of hypopneic events among the remaining AHI events.
- 24-hour ambulatory blood pressure monitoring (ABPM) was taken into consideration in two studies. In one of these studies there was not a statistically significant difference compared to baseline with the OA [30], while in the other study [20] the median diastolic pressure reduced significantly through the OA in 24 hours (the systolic pressure did not show this difference). Moreover, in this study the daily systolic and diastolic pressure significantly decreased compared to baseline. This was not the case for the pressure values while sleeping. In a secondary RCT [48], the change in night blood pressure which was statistically significant only decreased in women.
- Self-reported compliance was generally high for the number of nights when the device had been used and the sleep time for each night spent with the oral appliance. MAD reported that compliance was always higher than the CPAP compliance. Only in two RCTs [28, 30], a microsensor was used for a better compliance assessment in OA treatment. A study [30] showed a strong correlation between the sensor-reported compliance percentage and the self-reported compliance percentage (96.1% vs 100%) in the MAD group treatment. This result did not apply to the sham device used in the other group of this study (in this case, the difference between objective compliance and self-reported compliance was huge). The other study [28] showed a lack of statistically significant difference between the percentage of compliance for OA and the positional treatment.

Among the 17 studies, there was only one that compared positional treatment with OA [28]. This RCT used a band with a vibrating device around the chest. Results showed that there was not a statistically significant difference in slight or moderate positional OSA (no patients with AHI >20) in terms of the AHI improvement. It was important to notice that nonsupine AHI was only controlled in OA treatment group while it was significantly increasing compared to the baseline in the positional treatment group. For these reasons, the combination of these two therapies could be a successful choice in selected patients. Another important consideration from this study is that in OA treatment group there was not a statistically significant difference in terms of the time spent in supine position (being opposite to positional treatment group) [28].

Self-reported data on symptoms and life quality [Epworth sleepiness score (ESS), snoring symptoms inventory (SSI), life quality (SF-36, FOSQ, SAQLI, etc.,)] showed rather variable results:

- SSI has been taken into consideration in only a study [21] showing a statistically significant reduction without differences in both analysed groups. In this study, the mandibular advanced device was nontitratable and was compared to a bite raising splint. From the snoring evaluation made by the partner [26], the lack of changes in snoring with placebo was more often reported. MAD increasingly showed a reduction in snoring and, more frequently, snoring disappeared completely with CPAP. In Marklund et al. [34], the oral appliance allows a statistically significant snoring reduction compared to placebo.
- The only study that took into consideration restless legs symptoms outlined a reduction which is statistically significant with OA compared to placebo [34].
- ESS (the most common subjective evaluation of sleepiness in the literature) is always statistically significantly better, but in the previously described study by Lam et al. [20] this reduction was also statistically significant in the placebo group, suggesting the possibility of a so-called placebo effect. Moreover, in another study there was not a statistically significant difference compared to placebo with an objective evaluation of sleepiness through the OSLER test [34] during OA treatment. The data from Benoist et al. [28] confirm the importance of an objective evaluation of sleepiness and outline the fact that even if there is a lack of statistically significant differences in the two therapies (oral appliance vs sleep position trainer or SPT) in terms of polysomnographic parameters' improvement, the change in ESS was statistically significantly reduced in the OA group compared to that of the SPT group;
- Life quality is evaluated in multiple studies through different types of questionnaires, and the results seem to be heterogeneous enough, but they do not provide a clear view on the outcome efficacy through OA treatment. It seems that the results of the only RCT [40] that is based on the Beck Depression Inventory and the Vigor-Activity and Fatigue-Inertia scale of the Proﬁle of Mood States, outline a significant improvement of fatigue, level of energy, and surveillance after OA treatment.

Among the studies with an overall low risk of bias, none could provide information on the success predictive factors of the treatment [25, 26]. Among the other studies, the one by Hoekema et al. [33] was the only RCT that led to a different publication from the one published in 2008 [41], a single and multivariate analysis in the two groups (MAD and CPAP treatment) with the aim of identifying potential predictive polysomnographic and/or cephalometric success therapeutic factors. This study outlined the lack of reliable predictive variables in the CPAP treatment while showing the following predictive factors of efficacy in OA treatment:

- Less obese patients (lower BMI)
- Patients with a less serious apnoea (lower respiratory indices)
- Patients with mandibular retrognathism
- Patients with the largest mandibular protrusion

An efficient OA treatment in the resolution of OSA can be a first choice in moderate-slight OSA patients. In these patients, the choice to use these devices can be determined by the previously listed predictive factors [41]. The evaluation of some of these predictive factors falls within the preparation of a dentist majored in sleep dentistry. For this reason, these patients should always be referred to the latter for an appropriate visit or analysed in a team led by this person. Positive effects of OA treatment should be further analysed in other studies on blood pressure which will be necessary to clarify, understand, and quantify them in OA treatment. Compliance data confirm excellent compliance values in OA treatment but strongly recommend the use of objective recording systems for this outcome. After proving the OA therapeutic efficacy in a patient, monitoring its use during the treatment will also be important considering the huge risks due to a lack of treatment compliance, both to one's health (CV risk, etc.) and to others' health (road accidents). Correct treatment adherence would bring benefits to national health savings due to the reduced need to treat all the potentially serious chronic OSA diseases or those diseases in which OSA is a concause.

In those cases of severe OSA where the OA is used for those patients refusing other treatments, the potential results that can be obtained should not be underestimated. From the current evidence, there is a substantial improvement in oxygen desaturation index (ODI) and apnoea index (AI). For this reason, even if the disease is only partially controlled through the OA (partial AHI success), it is clear that this treatment represents a fundamental choice to improve the life of patients with severe OSA that refuse other types of treatments. However, this is still a compromise for a patient's health. These considerations cannot be applied to those people who have to drive means of transport because, depending on the rate of partial success of the treatment, there could be damage to one's health or others' health. For these reasons, the OA application in specific severe OSA cases is still a valid choice compared to a lack of treatment. Considering the available evidence, the OA appears to be a solution for snoring more often than CPAP, but it is not able to eliminate it completely as it is the case for the latter. However, CPAP leads to increased levels of noise while sleeping and, as snoring, can become a reason of disturbance to the partner's sleep.

Subjective reported daily sleepiness through ESS outlines a statistically significant improvement following OA treatment, but some articles lack statistically significant differences compared to placebo. In the only study [34] in which sleepiness was objectively evaluated (OSLER test), there were no statistically significant differences compared to placebo. In the light of these results, sleepiness cannot be a predictive success factor and its improvement cannot be considered a success finding. In light of the aforementioned results, all the future efficacy studies and the predictive factors in OSA treatment should always use the best oral appliances (customised and titratable). Banhiran et al. [19] provide another source of reflection for potential improvements that can be implemented in future research considering the most recent recommendations in this field [6]. In this study, the customised oral appliance was also made and titrated by a non-dental health professional practitioner.

This analysis outlines another useful consideration for the daily clinical dentistry practice in OSA treatment. A lower-quality study [31] compared two customised titratable devices with two different titration systems: one of them (Herbst-like) with a titration system (telescopic arms) in the splint sides and the other (TAP) with a titration system (screw) in the front. The front activation system of the device also allowed the mandible to be closed during the night. Ghazal et al. [31] carried out a short and long term follow-up. The results show that the device with the titration system in the front (provided with a “front connector”) turned out to be statistically significantly more efficient in reducing respiratory indices and ODI in the short term compared to the one with side titration system (the one without a “front connector”).

As shown, in a chronic disease such as OSA, reaching therapeutic efficacy is one of the factors that affect compliance and treatment adherence. In a recent study [49] on long term adherence in OA treatment for OSA, the authors found that the therapeutic efficacy is the most important factor (100% of the cases) and is associated with continued treatment. Moreover, one of the selected RCTs [30], in which compliance was objectively estimated, outlined a statistically significant difference between a placebo splint and an active MAD, favouring the latter. Available data confirm that the level of compliance can be potentially reduced due to a lack of efficacy in the treatment.

### 4.1. Limitations

The main limitations of this systematic review were (a) a median quality of the selected studies which is not high; (b) different types of oral appliances; (c) different types of placebo; (d) different follow-up duration; and (e) different range of respiratory indices of severity among the inclusion criteria.

## 5. Conclusions

The present systematic review of the oral appliance treatment in patients with mild-to-moderate OSA suggests some evidence for cases of severe OSA. From a public health view, it is very urgent to bridge the gap between the epidemiological relevance of OSA, the health consequences, and the low average quality of evidence on the treatment effectiveness proposed by the scientific community. A comprehensive approach based on studies with longer follow-up periods (including the creation of population-based pathology records to assess the predictive variables on the treatment effectiveness in the medium and long period) will improve the quality of evidence in this field and establish more rigorous guidelines that will need to be promoted through appropriate training and an information strategy targeting field professionals.

## Figures and Tables

**Table 1:** Clinical and demographical characteristic of the 17 RCTs included.

Included RCT (country where the study was conducted)	RCT design (treatments compared with OA)	Sample size diagnostic procedure (AHI/RDI)	Type of oral appliance	Follow-up period	Main conclusion (primary outcome)
Aarab et al. [26] (Netherlands)	Parallel RCT (CPAP; placebo)	64 (47 M and 17 F; mean age: 50.3 ± 9.1 yrs in MAD group, 55.4 ± 9.8 yrs in nCPAP group, and 51.3 ± 10.1 yrs in placebo group); full polysomnographic recordings in the sleep laboratory of the Slotervaart Medical Centre, using Siesta hardware and Profusion software (Compumedics, Abbotsford, VIC, Australia) (5 ≥ *e* ≤ 45)	Customised and titratable device	6 ± 2 months	No differences in the AHI were found between the MAD and nCPAP therapy (*p*=0.092), whereas the changes in AHI in the two therapy groups were significantly greater than those in the placebo group (*pp* < 0.001 and *p*=0.002, respectively)

Andrén et al. [25] (Sweden)	Parallel RCT (placebo)	72 (57 M and 15 F; mean age: 57 ± 8 yrs in active OA group and 59 ± 9 yrs in control OA group); ambulatory nocturnal somnographic registration (Embletta PDS device; Medcare Flaga, Iceland) (≥10)	Customised monobloc nontitratable device	3 months	Significant AHI reduction in patients with active OA (*p* < 0.001). Significant 24 h mean systolic blood pressure reduction was noted only in a subgroup of patients with ambulatory 24 h mean systolic BP > 135/85 mmHg and AHI >15

Banhiran et al. [19] (Thailand)	Crossover noninferiority RCT (CPAP)	50 divided into two groups of 25, with mean age of 47.1 ± 11.0 yrs (group A) and 52.2 ± 9.8 yrs (group B); home WatchPAT monitoring (≥5)	Titratable but not customised device (not adapted and managed by a dentist)	1.5 months	There was no statistically significant difference in all dimensions of FOSQ scores between CPAP and AT-MAS

Barnes et al. [27] (Australia)	Crossover RCT (CPAP; placebo)	80 (mean age of 46.4 yrs, with 78.8% of the subjects being male); polysomnography (between 5 and 30)	Customised and titratable device	3 months	Both CPAP and MAS improve AHI and night hypoxia in a statistically significant way compared to placebo. The results were better with CPAP than MAS

Benoist et al. [28] (Netherlands)	Parallel RCT (positional therapy)	99 (70 M and 29 F; mean age: 47.3 ± 10.1 yrs in SPT group and 49.2 ± 10.2 yrs in OA group); a digital PSG system (Embla A10, Broomfield, CO, USA) (positional OSAS)	Customised and titratable device	3 months	There was no statistically significant difference in AHI reduction between the two groups

Gagnadoux et al. [29] (France)	Crossover RCT (CPAP)	59 (46 M and 13 F; mean age: 50.3 ± 9.1 yrs); in-laboratory PSG (CID 102 TM; Cidelec) (between 10 and 60)	Customised and titratable device	2 months	Median AHI value (interquartile range) was 2 [1–8] with CPAP *e* 6 [3–14] with MAD (*p* < 0.001)

Gagnadoux et al. [30] (France)	Parallel RCT (placebo)	150 (14.4% were female; mean age: 54.8 ± 9.9 yrs in MAD group, 52.9 ± 10.5 yrs in sham device group); in-laboratory PSG (≥30)	Customised and titratable device	2 months	After adjustment for baseline values, age, sex, BMI, AHI, and smoking habits, the difference in RHI outcome between effective MAD and sham device was not statistically significant

Ghazal et al. [31] (Germany)	Parallel RCT (other customised and titratable MAD)	103 (ST group mean age: 50.5 ± 10.9 yrs, 41 M, 10 F; TAP group mean age: 50.4 ± 11.1 yrs, 45 M, 7 F); polysomnography (PSG) (≤40)	Customised and titratable device	Median time interval of follow-up: 42.7 months for IST appliance; 41.5 months for TAP appliance	Significant reduction of AHI was noticed with both the devices. In the short-term evaluation, the device that held the mandible firmly in a protrusive position during the entire sleep (TAP), without allowing mouth opening, was significantly better than the other one (IST)

Gotsopoulos et al. [32] (Australia)	Crossover RCT (placebo)	73 (59 M, 14 F; mean age: 48 ± 11); polysomnography (PSG) (≥10)	Customised and titratable device	1 month	Both MLST and ESS values were better at follow-up. A significant reduction of the following values in MAS group with respect to control group was noticed: AHI, snoring frequency, mean and maximum snoring intensity, arousal index, and MinSaO_2_

Hoekema et al. [33] (Netherlands)	Parallel noninferiority RCT (CPAP)	103 (92 M and 11 F); polysomnography (Embla® A10 digital recorder, Medcare, Reykjavík, Iceland) (≥5)	Customised and titratable device	3 months	Noninferiority of oral appliance therapy was considered to be established when the lower boundary of this interval exceeded −25%. The lower boundary of the confidence interval was −21.7%, indicating that oral appliance therapy was not inferior to CPAP for effective treatment of obstructive sleep apnoea. However, subgroup analysis revealed that oral appliance therapy was less effective in individuals with severe disease (apnoea-hypopnea index >30)

Lam et al. [20] (Hong Kong)	Parallel RCT (CPAP; conservative measure)	101 (79 M and 22 F); PSG (Alice 3 or Alice 4 Diagnostics System, Respironics, Atlanta, USA) (between 5 and 40)	Customised but not titratable device	2.5 months	Nadir O_2_ and AHI improved significantly with respect to baseline values both in CPAP group and in OA group. ESS significantly decreased in all three groups

Maguire et al. [21] (United Kingdom)	Crossover RCT (placebo)	52 (36 M and 16 F, mean age: 44.6 yrs); diagnostic procedure not specified (≤15)	Customised but not titratable device	3.5 months	ESS and SSI values reduction of MAS versus BRA was not statistically significant

Marklund et al. [34] (Sweden)	Parallel RCT (placebo)	91 (62 M and 29 F; mean age: 49.8 ± 10.6 yrs in OA group and 54.1.2 ± 9.4 yrs in placebo device group); polysomnographic sleep recordings (Embla, Natus Neurology) (<30)	Customised and titratable device	4 months	No significant difference for the primary outcomes (ESS, KSS, OSLER test, SF-36) between the two groups

Phillips et al. [35] (Australia)	Crossover noninferiority RCT (CPAP)	126 (102 M and 24 F; mean age: 49.5 ± 11.2 yrs); polysomnography (PSG) (>10)	Customised and titratable device	1 month	MAD was noninferior to CPAP for control of 24MAP (mean CPAP-MAD difference (95% confidence interval), 0.2 (20.7 to 1.1) mm Hg). In the subgroup of patients who were initially hypertensive, there were consistent treatment-related 24-hour BP improvements of 2–4 mm Hg in all indexes with neither treatment having a superior effect

Quinnel et al. [22] (United Kingdom)	Crossover RCT, no treatment; thermoplastic “boil and bite” not titratable device (SleepPro 1); semi-bespoke not titratable device produced from a patient-moulded dental impression kit (SleepPro 2)	90 (72 M and 18 F; mean age: 50.9 ± 11.6 yrs); respiratory polysomnography (rPSG) (5≥ and ≤30)	Customised but not titratable bespoke MAD (bMAD)	1 month	All three MADs significantly decreased the AHI against no treatment by 26% (95% CI: 11% to 38%) for the SP1, 33% (95% CI: 24% to 41%) for the SP2, and 36% (95% CI: 24% to 45%) for the bMAD. A similar effect was found for all devices against no treatment for 4% oxygen desaturation index (4% ODI). The bMAD had a significant effect on minimum oxygen saturation compared with no treatment and the other devices

Tegelberg et al. [23] (Sweden)	Parallel RCT (same OA with a different degree of advancement)	74 (mean age: 51, 8 yrs in 50% group and 54.4 yrs in 75% group); home sleep study using a portable unit (5≥ and ≤25)	Customised monobloc nontitratable device	12 months	Significant reduction of AI, AHI, and ODI in both groups (group with 50% of advancement and group with 75% of advancement)

Wilhelmsson et al. [24] (Sweden)	Parallel RCT (UPPP surgical procedure)	95 (mean age: 49, 3 yrs in OA group and 51 yrs in UPPP group); somnography (>25)	Customised monobloc nontitratable device	12 months	Significant reduction of AI, AHI, ODI, and SI in both groups at 6 and 12 months. After 12 months, OA gave better results than UPPP. Success rate for AI and AHI resolution with OA was, respectively, 95% and 81%. Success rate for AI and AHI resolution with UPPP was, respectively, 70% and 60%

**Table 2:** Cochrane Collaboration's tool for assessing risk of bias for the 17 identified RCTs.

Studies	Random sequence	Allocation concealment	Blinding of participants and personnel	Blinding of outcome assessment	Incomplete outcome data	Selective reporting	Other bias	Overall risk of bias
Aarab et al. [26]	Low	Low	Low	Unclear	Low	Low	Unclear	Low risk
Andrén et al. [25]	Low	Low	Unclear	Low	Low	Unclear	Unclear	Low risk
Banhiran et al. [19]	High	High	High	High	Low	Low	Unclear	High risk
Barnes et al. [27]	Low	High	High	High	Low	Unclear	Unclear	High risk
Benoist et al. [28]	Low	Unclear	High	High	Unclear	Low	Unclear	High risk
Gagnadoux et al. [29]	High	High	High	High	Unclear	Unclear	Unclear	High risk
Gagnadoux et al. [30]	Low	High	High	High	Unclear	Low	Unclear	High risk
Ghazal et al. [31]	Low	High	High	High	Unclear	Unclear	Unclear	High risk
Gotsopoulos et al. [32]	Unclear	Unclear	Low	High	High	High	Unclear	High risk
Hoekema et al. [33]	Low	High	Unclear	Unclear	Low	Low	Unclear	High risk
Lam et al. [20]	Unclear	Unclear	Unclear	Unclear	Unclear	Unclear	Unclear	Unclear risk
Maguire et al. [21]	Low	Low	Unclear	Unclear	High	Unclear	Unclear	Unclear risk
Marklund et al. [34]	Low	Unclear	Low	High	High	High	Unclear	High risk
Phillips et al. [35]	Unclear	Unclear	Unclear	Unclear	Low	Low	Unclear	Unclear risk
Quinnel et al. [22]	Low	Unclear	High	Unclear	High	High	Unclear	High risk
Tegelberg et al. [23]	Low	Low	Unclear	Unclear	Low	Unclear	Unclear	Unclear risk
Wilhelmsson et al. [24]	Low	Low	Unclear	Unclear	Low	Unclear	Unclear	Unclear risk

## Data Availability

The datasets used and/or analysed during the current study are available from the corresponding author on reasonable request.

## Abbreviations

OSA: Obstructive sleep apnoea
RCT: Randomised clinical trial
OSAS: Obstructive sleep apnoea syndrome
AHI: Apnoea-hypopnoea index
CPAP: Continuous positive airway pressure
OA: Oral appliances
RDI: Respiratory disturbance index
ESS: Epworth sleepiness score
AASM: American Academy of Sleep Medicine
AADSM: American Academy of Dental Sleep Medicine
MAD: Mandibular advancement device
BMI: Body mass index
ABPM: 24-hour ambulatory blood pressure monitoring
SPT: Sleep position trainer
ODI: Oxygen desaturation index
AI: Apnoea index
PSG: Polysomnography.
